# Embedding a primary care provider in sickle cell teams improves sickle cell care

**DOI:** 10.1371/journal.pone.0352670

**Published:** 2026-06-25

**Authors:** Sherraine Della-Moretta, Rina Li Welkie, Nives Quaye, Kristina Landes, Regina D. Crawford, Payal Desai, Robert M. Cronin

**Affiliations:** 1 Department of Internal Medicine, The Ohio State University, College of Medicine, Columbus, Ohio; 2 Levine Cancer Institute, Advocate Health, Wake Forest School of Medicine Charlotte, Charlotte, NC; Versiti Blood Research Institute, UNITED STATES OF AMERICA

## Abstract

Adults with sickle cell disease (SCD) experience fragmented access to primary and preventive care, which leads to poor adherence to general and SCD‑specific clinical practice guidelines. To address this gap, we implemented an embedded primary care model where a board‑certified internist/pediatric primary care provider (PCP) was embedded as a full member of the adult SCD care team, attending operational and educational meetings and practicing alongside hematologists in a comprehensive SCD clinic. Our primary aim was to test the hypothesis that an embedded primary care model was associated with improved guideline‑based preventive care and changes in acute healthcare utilization (e.g., emergency room visits and hospitalizations). We conducted a retrospective cohort study of adults with SCD seen at a single tertiary care center between July 2020 and June 2025. Of the 388 adults with SCD seen at the center, 174 received care from the embedded PCP. Patients in the embedded PCP model demonstrated significantly higher adherence to general preventive care including cervical cancer screening ((Odds Ratio) OR: 4.49; 95% (Confidence Interval) CI: 2.46, 8.23), depression screening (OR: 7.97; 95% CI: 1.78, 35.69), and diphtheria-tetanus-pertussis (Tdap) immunization (OR: 2.88; 95% CI:1.72, 4.84), and SCD‑specific guidelines, including annual eye examinations (OR: 2.59; 95% CI: 1.66, 4.04), pneumococcal immunization (OR: 3.58; 95% CI: 2.16, 5.92), urine protein screening (OR: 3.36; 95% CI: 1.89, 6.00), and ACE inhibitor/ARB use for microalbuminuria (OR: 9.37; 95% CI: 3.11, 28.23). Among patients who saw the embedded PCP, patients had significantly more annual outpatient visits (post-PCP: 4.2 vs pre-PCP: 2.7, p < 0.0001) and, while insignificant, fewer annual inpatient admissions (post-PCP: 1.4 vs pre-PCP: 1.9, p = 0.4869). Embedding a PCP within the adult SCD care team was associated with improved guideline‑based preventive care and more annual outpatient visits, which was consistent with more coordinated, outpatient‑focused management.

## Introduction

Sickle cell disease (SCD) is an inherited condition of hemoglobin affecting 100,000 individuals in the United States. Individuals with SCD primarily comprise socially disadvantaged groups that experience disparities in care and treatment [1–7]. Asthma, mental health, women’s health issues, and headaches are common primary care problems in the general population treated by primary care providers (PCPs) [8–14]. There is a high prevalence and importance of chronic pain [15,16], asthma [17–21], depression [22–24] women’s health [25–27], and headache control [28] in SCD. However, these issues are typically unrecognized or managed by the hematologist untrained in these primary care issues due to the lack of PCPs. Also, if these issues are not recognized and adequately managed, each can negatively impact SCD management and outcomes. National guidelines have been developed for preventive care to improve health and reduce the burden of disease. Some of these guidelines are for the general public, such as cancer, diabetes, mental health, and sexually transmitted infection screening. Additionally, the American Society of Hematology has published guidelines that include preventive care for neurocognitive, cardiopulmonary, and kidney disease [29,30]. The National Alliance of Sickle Cell Disease Centers released more extensive consensus recommendations on SCD health maintenance including organ-specific screening in addition to preventive and reproductive counseling [31]. Therefore, to be able to prevent and treat disease, individuals with SCD require a team approach, with both PCPs trained in primary care issues and hematologists trained in SCD.

Community PCPs willing to accept adults with SCD are challenging to find, are typically unknowledgeable about SCD, have negative attitudes about SCD, including stigma and mistrust about pain [32–37], and access to primary care may be reduced for patients taking prescription opioids [38,39]. Individuals with SCD also report many access-level barriers to PCPs, including difficulty contacting their doctors and clinics, waiting an extended time to see a provider, or only able to access care during inconvenient clinic hours. Individuals with SCD who can access care still report challenges to adequate care, including having unknowledgeable PCPs, poor patient-provider relationships, uncoordinated care, and that they experience stigma, physician mistrust, and care that is not focused on their wellbeing [32–34,40,41]. With these challenges, there is a gap in our understanding of how best to deliver both SCD-specific and primary care for this population.

Prior studies suggest that primary care involvement may improve outcomes for adults with SCD [42–44]. A recent concise report described an adult comprehensive SCD center integrating same-day primary care within multidisciplinary visits and evaluated ED utilization and selected preventive screenings over a shorter observation window [43]. Additionally, a multi-system analysis of adults with SCD found that “shared care” (having both a PCP and a hematologist) was associated with lower likelihood of frequent hospitalizations, though not ED use [42]. However, there remains limited evidence describing (1) longer-term implementation of a co-located integrated PCP model embedded in an adult SCD teams and (2) its association with a broad set of USPSTF preventive services and SCD-specific guideline adherence.

An embedded PCP in an SCD clinic can address and manage preventive care and primary care issues. Our embedded PCP model is defined as a PCP board-certified in pediatrics and internal medicine who delivers primary care to adults with SCD, embedded and co-located within the pediatric and adult clinics and teams that provide subspecialty SCD care for these patients. Adults with SCD are not responsible for identifying and seeking a community PCP, nor do SCD providers need to refer their adults with SCD to adult community PCPs. The embedded PCP provided essential primary care during their SCD specialist visits and managed primary care. The PCP participated in the SCD team’s educational and quality-improvement activities to maintain knowledge and remain up to date on SCD care. Therefore, the embedded PCP delivered care in accordance with the premise of Wagner’s Chronic Care Model: a prepared, proactive clinical practice team within the pediatric and adult SCD clinics that is integral to achieving an effective Patient-Centered Medical Home. As part of the multidisciplinary team, a nurse case manager or social worker implemented the social components of the biopsychosocial model for all patients; a hematologist provided care for SCD; and the PCP addressed the additional biological, psychological, and social components and coordinated care and interacted with all other team members for the embedded PCP group.

To measure the effectiveness of the embedded PCP in a SCD clinic, this study aimed to evaluate the differences in preventive care that adults with SCD received, emergency department (ED) visits, and inpatient hospital admissions. We tested the hypotheses that adults with SCD who were seen in the embedded PCP clinic would (1) have higher rates of guideline-based primary and SCD-specific care, (2) have higher annual rates of outpatient visits, and (3) have lower annual inpatient hospitalizations and readmissions than those who were not seen in the embedded PCP clinic.

## Methods

### Overview

This was a retrospective chart review study of a quality improvement project to deliver optimal care for adults with SCD through a patient-centered medical home with an embedded PCP at the Ohio State University Wexner Medical Center Comprehensive Sickle Cell Clinic. All patients who were seen in the SCD clinic between July 2020 and June 2025 were included. Data was accessed between September 1, 2025, and February 1, 2026, and authors had access to information that could identify individual participants during or after data collection. As part of a quality improvement initiative, we developed dashboards to evaluate the percentage of patients who were following the different preventive health guidelines, including general, primary care and SCD-specific.

### Embedded PCP

In July 2020, The Ohio State University Wexner Medical Center Comprehensive Sickle Cell Clinic, developed an embedded primary care model. In this model, a PCP, would see patients alongside a hematologist in the same clinic. Adults over 18 years old with any type of SCD who did not have a PCP were offered the services of the embedded PCP in clinic. The embedded PCP delivered essential clinical primary care during their SCD specialist visits, including both general preventive care (e.g., cancer screening) and SCD-specific preventive care (e.g., annual urine protein checks). The embedded PCP was a full-time provider but spent only part-time, or 1 full clinic day (8 hours) per week, in the SCD clinic with the hematologists and other team members. The embedded PCP had support from nursing and a social worker when seeing SCD patients, although sometimes patients would not see the hematologist when seeing the PCP in a clinic visit. The embedded PCP was available by phone or message at any time, during or outside of the clinic. The PCP’s training included board certification in internal medicine and pediatrics, but there was no formal training in SCD. The PCP participated in the SCD team’s educational and quality-improvement activities to remain knowledgeable and up to date on SCD care. Specifically, the PCP was present at a weekly census meeting to discuss patients and at monthly meetings for QI and other education. Therefore, the embedded PCP delivered care through a prepared, proactive clinical practice team within the pediatric and adult SCD clinics, which is integral to achieving an effective Patient-Centered Medical Home.

### Guideline adherence

We used our health maintenance categories in our electronic health record system, Epic, to extract the guideline adherence. Many health maintenance categories were predetermined based on USPSTF guidelines A and B recommendations (https://www.uspreventiveservicestaskforce.org/uspstf/recommendation-topics/uspstf-a-and-b-recommendations) or CDC recommendations for vaccinations. We also built additional topics for SCD-specific guidelines from prior American Society of Hematology (ASH) [45] and National Heart, Lung, and Blood Institute (NHLBI) guidelines [46]. Patients who were eligible for the guideline were included in both the denominator and the numerator for the number who completed the guideline. Some participants would not be eligible for certain guidelines or may have different frequencies depending on their demographics or prior results. For example, a 25-year-old would not be due for colorectal screening, and the next cervical cancer screening would depend on the patient’s age and previous pap smear and HPV testing results. Therefore, the necessity for the health maintenance category to be fulfilled and frequency would depend on the USPSTF, CDC, ASH, or NHLBI guidelines and may have different health maintenance topics within Epic (S1 File). The Status values were categorized into the labels used for reporting as shown below: (1) “Up to Date”: “Not Due”, “Due Soon”, “Completed”, or “Addressed”; (2) “Needs to Be Completed”: “Due On”, “Overdue”, or “Postponed”; (3) “Excluded”: “Aged Out”, “Discontinued”, or “Hidden” (S2 File). We defined the % complete as the distinct count of “Up to Date” patients divided by the distinct count of “Up to Date” or “Needs to Be Completed” patients. Those who were “up to date” were counted in the numerator, “Up to Date” and “Needs to be Completed” were denominator and “Excluded” were removed from analysis. Most recent completion value was used in reporting. The ACE/ARB category was based on the number of participants who had had an ACE or ARB prescribed within the year prior to the final visit, out of the total number of participants who ever had an elevated albumin to creatinine ratio over 100. All the guideline adherence outcomes were determined to be “up to date” based on the last encounter date, therefore in the case of annual protein screening, the “up to date” status would be based on whether the screening was complete completed during the prior year.

### Acute healthcare utilization

All-cause ED visits and all-cause inpatient hospitalizations were assessed through electronic health record (EHR) chart review for all adults with sickle cell disease included in the study. For the utilization analysis, we included participants who had at least 2 visits and at least 365 days of follow-up. For participants in the embedded PCP group, ED visits and hospitalizations were measured from the date of the first visit with the embedded PCP through the date of the most recent recorded visit. For participants who did not receive embedded PCP care, ED visits and hospitalizations were measured from the date of their first recorded clinical encounter within the study period through their last recorded encounter. The total number of ED visits and hospitalizations during these observation periods were extracted and used as indicators of acute care utilization. These numbers were divided by the total amount of time (in years) and represented as average annual acute care utilization. Readmissions were measured as repeated inpatient hospitalizations within 30 days of the last inpatient hospitalization.

### Statistical analysis

Descriptive statistical analyses were conducted to describe aspects of the population. Baseline categorical characteristics were summarized using frequencies and percentages and compared using the Chi-square test, or Fisher’s Exact test when expected counts are equal to or less than 5. Continuous measurements were summarized as median and range and compared using the Wilcoxon rank-sum test. The guideline outcomes at the most recent visit were analyzed using logistic regression, adjusting for age at the visit, sex, race, and SCD type. Poisson regression and Poisson generalized estimating equation (GEE) model also adjusted for age at the visit, sex, race, and SCD type, which accounted for repeated subjects, was applied to compare hospital utilization data between non-PCP and PCP groups and before and after seeing the PCP. All analyses were conducted using SAS (version 9.4).

### Human ethics and consent to participate declarations

**Fig 1:**
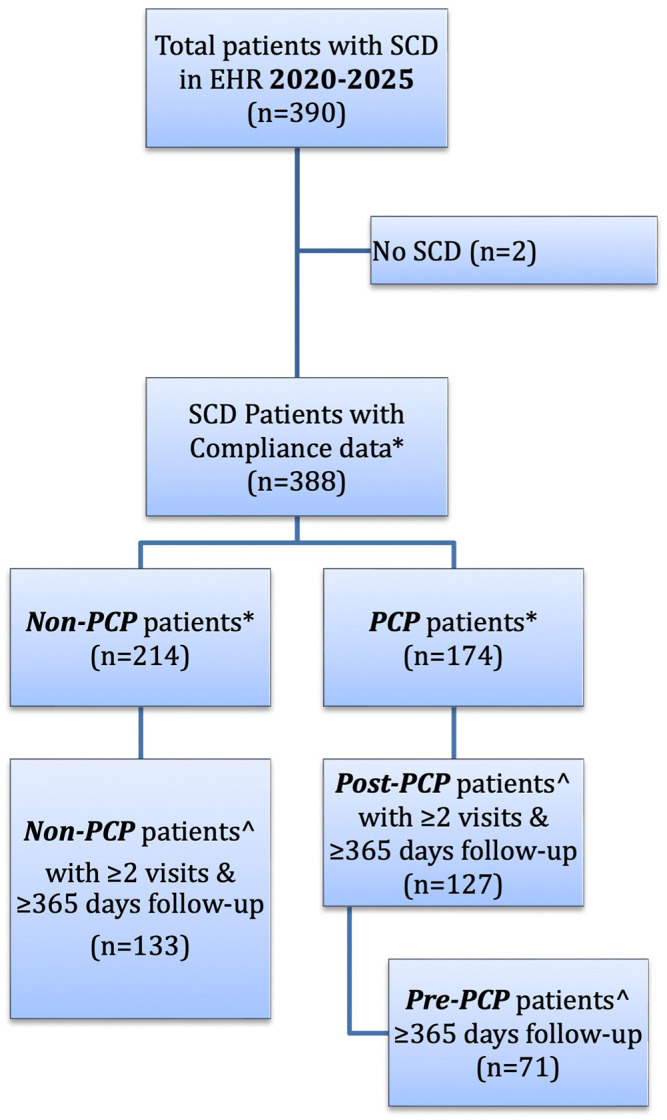
Flow diagram of patients. *: Table 1 and Fig 2; ^: Tables 2 and 3.

This retrospective study was approved by the Ohio State University Institutional Review Board, with a waiver of informed consent Fig 1.

## Results

A total of 388 adults with SCD were seen in the SCD clinic, of which 174 adults were seen by the embedded PCP (Table 1). The median age was 34 years (Interquartile range (IQR): 18–75 years); 59.8% were female, and the majority were Black (94.9%).

**Table 1:** Patients’ Demographics at last visit, n= 388.

Characteristics	Total (n = 388)	Non-PCP (n = 214)	PCP (n = 174)	P-value
Age at last visit, median (range)	34 (18, 75)	35 (18, 75)	34 (19, 70)	0.139
Gender, n (%)				0.143
Female	232 (59.79)	135 (63.08)	97 (55.75)	
Male	156 (40.21)	79 (36.92)	77 (44.25)	
Race, n (%)				0.989
Non-Black	20 (5.15)	11 (5.14)	9 (5.17)	
Black	368 (94.85)	203 (94.86)	165 (94.83)	
SCD type, n (%)				0.448
SS/S-beta0	195 (50.26)	113 (52.80)	82 (47.13)	
SC/S-beta+	190 (48.97)	100 (46.73)	90 (51.72)	
Other	3 (0.77)	1 (0.47)	2 (1.15)	
Insurance health coverage, n (%)				0.383
Private	166 (42.78)	97 (45.33)	69 (39.66)	
Public	213 (54.90)	111 (51.87)	102 (58.62)	
Other	9 (2.32)	6 (2.80)	3 (1.72)	

### Patients who saw the embedded PCP had better USPSTF guideline adherence than those who did not see the embedded PCP

**Fig 2:**
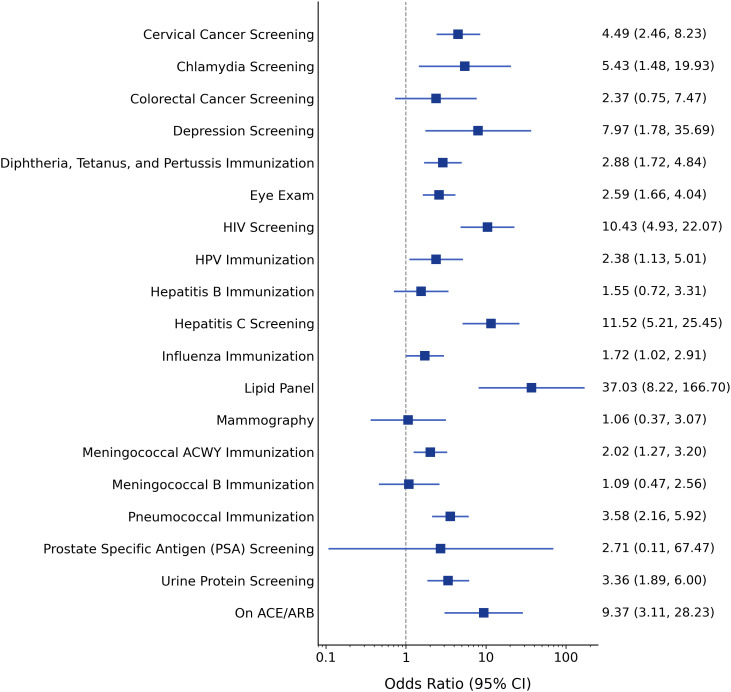
Forest plot of adherence to guidelines of the embedded PCP vs. no embedded PCP. An Odds Ratio > 1 is increased likelihood of guideline adherence with the embedded PCP.

Participants who received care from the embedded primary care provider (PCP) demonstrated significantly higher adherence to multiple U.S. Preventive Services Task Force (USPSTF) guideline–recommended screenings and immunizations compared with those who did not (Fig 2, S3 File). Specifically, patients in the embedded PCP group were more likely to have received cervical cancer screening (Odds Ratio (OR): 4.49; 95% Confidence Interval (95% CI): 2.46, 8.23), chlamydia screening (OR: 5.43; 95% CI: 1.48, 19.93), depression screening (OR: 7.97; 95%CI: 1.78, 35.69), diphtheria-tetanus-pertussis (Tdap) immunization (OR: 2.88; 95% CI:1.72, 4.84), HIV screening (OR: 10.43; 95% CI: 4.93, 22.07), hepatitis C screening (OR: 11.52; 95% CI: 5.21, 25.45), and lipid panel testing (OR: 37.03; 95% CI: 8.22, 166.74). Other USPSTF guideline rates did not differ significantly between groups.

### SCD-specific guideline adherence was better with an embedded PCP

Adherence to SCD-specific care, as recommended by the ASH and NHLBI guidelines and NASCC consensus recommendations, was significantly higher among participants who received care from the embedded PCP than among those who did not (Fig 2, S3 File). Patients in the embedded PCP group were more likely to have completed annual eye exams (OR: 2.59; 95% CI: 1.66, 4.04), meningococcal ACWY immunization (OR: 2.02; 95% CI: 1.27, 3.20), pneumococcal immunization (OR: 3.58; 95% CI: 2.16, 5.92), and urine protein screening (OR: 3.36; 95% CI: 1.89, 6.00). Rates of *Haemophilus influenzae* type b (Hib) and meningococcal B immunizations were similar between groups. Among patients with microalbuminuria, over double in the embedded PCP group had a prescription for an ACEi/ARB in the past year (OR: 9.37; 95% CIΩ:3.11, 28.23). Our findings from a sensitivity analysis requiring 2 visits and 365 days or more of follow up are presented in S4 File.

### Adults in the embedded PCP group had significantly higher rates of outpatient visits after integration of the PCP

Before implementation of the embedded PCP model, adults in the PCP group did not have any significant differences in annual outpatient visits, admissions, ED visits or readmissions compared with those not seen by the embedded PCP (Table 2). After introduction of the embedded PCP, outpatient visit frequency increased among PCP patients (median 4.2 vs. 2.8 visits/year, p < 0.0001), while ED visits were significantly higher than in the non‑PCP group (median 2.2 vs. 1.6 visits/year, p < 0.0001). No significant differences were observed post‑implementation in annual inpatient admissions,. Overall, patients followed by the embedded PCP had higher healthcare utilization both before and after integration, though several measures, particularly hospitalization metrics, showed attenuation in differences after integration (Table 2). Patients who saw the embedded PCP also demonstrated meaningful within‑group changes over time. After establishing care with the embedded PCP, patients had substantially higher annual outpatient visits (median before: 2.7 vs. after: 4.2 years, p < 0.0001). Annual admissions and readmissions remained statistically unchanged, although annual admission rates were lower (before: 1.9 vs. after:1.4, p = 0.4869) Table 3.

**Table 2:** Utilization Comparison between non-PCP and PCP patients (at least 2 visits and 365 days FU).

Outcomes	All Patients		Non-PCP			pre-PCP			P-value
	n	# of Visits, median (range)	n1	# of Visits, median (range)	Estimate (95% CI)	n2	# of Visits, median (range)	Estimate (95% CI)	
Years of Outpatient Follow-Up	204	3.1 (1.0, 5.1)	133	4.0 (1.0, 5.1)	3.37 (2.73, 4.15)	71	2.0 (1.0, 4.7)	2.36 (1.84, 3.02)	0.0001
Average Annual Visits of Outpatient	204	2.8 (0.2, 8.0)	133	2.8 (0.9, 8.0)	3.12 (2.55, 3.83)	71	2.7 (0.2, 6.4)	2.92 (2.31, 3.69)	0.4559
Average Annual Visits of Admission	138	1.3 (0.2, 11.3)	90	1.2 (0.2, 11.3)	2.67 (2.16, 3.30)	48	1.9 (0.3, 10.5)	2.88 (2.22, 3.75)	0.5118
Average Annual Visits of ED	164	1.7 (0.2, 59.4)	107	1.6 (0.2, 30.3)	4.74 (4.01, 5.60)	57	2.2 (0.2, 59.4)	5.47 (4.50, 6.63)	0.0662
Average Annual Visits of Readmission	52	0.8 (0.2, 8.2)	33	0.8 (0.2, 8.2)	1.14 (0.70, 1.87)	19	0.7 (0.2, 5.6)	1.26 (0.72, 2.20)	0.6806
Years of Outpatient Follow-Up	260	3.4 (1.0, 5.1)	133	4.0 (1.0, 5.1)	3.27 (2.73, 3.92)	127	3.0 (1.0, 4.7)	2.95 (2.45, 3.55)	0.1470
Average Annual Visits of Outpatient	260	3.6 (0.9, 8.3)	133	2.8 (0.9, 8.0)	3.00 (2.54, 3.55)	127	4.2 (1.5, 8.3)	4.53 (3.86, 5.31)	<.0001
Average Annual Visits of Admission	185	1.3 (0.2, 11.3)	90	1.2 (0.2, 11.3)	2.88 (2.39, 3.47)	95	1.4 (0.2, 9.6)	2.84 (2.35, 3.43)	0.8835
Average Annual Visits of ED	222	1.8 (0.2, 61.0)	107	1.6 (0.2, 30.3)	4.99 (4.38, 5.69)	115	2.2 (0.2, 61.0)	8.41 (7.49, 9.46)	<.0001
Average Annual Visits of Readmission	72	0.9 (0.2, 8.2)	33	0.8 (0.2, 8.2)	1.42 (0.96, 2.11)	39	0.9 (0.2, 8.1)	1.67 (1.17, 2.40)	0.3900

**Table 3:** Patients’ utilization comparison between pre-PCP and post-PCP visits.

Outcomes	pre-PCP			post-PCP			p-value
	n1	# of Visits, median (range)	Estimate (95% CI)	n2	# of Visits, median (range)	Estimate (95% CI)	
Years of Outpatient Follow-Up	71	2.0 (1.0, 4.7)	2.09 (1.85, 2.35)	127	3.0 (1.0, 4.7)	2.76 (2.54, 3.00)	0.0001
Average Annual Visits of Outpatient	71	2.7 (0.2, 6.4)	2.75 (2.52, 3.01)	127	4.2 (1.5, 8.3)	4.42 (4.14, 4.73)	<.0001
Average Annual Visits of Admission	48	1.9 (0.3, 10.5)	2.51 (1.68, 3.75)	95	1.4 (0.2, 9.6)	2.82 (1.98, 4.01)	0.4869
Average Annual Visits of ED	57	2.2 (0.2, 59.4)	4.69 (2.65, 8.31)	115	2.2 (0.2, 61.0)	8.62 (5.44, 13.64)	0.0041
Average Annual Visits of Readmission	19	0.7 (0.2, 5.6)	1.18 (0.72, 1.94)	39	0.9 (0.2, 8.1)	1.46 (0.93, 2.29)	0.4045

## Discussion

Our findings extend emerging evidence supporting primary care integration for adults with SCD by evaluating a co-located embedded PCP model in SCD care teams over a 5-year period and by assessing a broad range of USPSTF preventive services and SCD-specific guideline adherence, including vaccination, kidney screening, ophthalmologic screening, and ACEi/ARB use among those with microalbuminuria. In contrast to prior reports that focused on selected screenings and shorter follow-up windows [42,43], our data provide a longer-term view of preventive care completion and evolving utilization patterns after establishing embedded primary care. Others have also demonstrated the decrease in co-management as individuals with SCD get older [47], making a model like this in adults critical. While there may be challenges in the development of such a model, the potential of improved care helps justify the need for this model. Future research demonstrating improvements in healthcare, utilization, and management of other chronic diseases, including pain, is needed. Also, prospective studies across multiple institutions are an area for further research.

Our embedded PCP was associated with more adherence to guidelines for general primary care than those who did not see the embedded PCP. We saw a higher level of adherence across a variety of USPSTF grade A and B recommendations. The most striking differences included sexually transmitted infection screening, immunizations, mental health screening, and women’s health. Others have demonstrated better adherence to guidelines using a multidisciplinary approach [43,48], but ours demonstrated significantly better adherence across many more guideline recommendations. The potential, long-term implications of better adherence to guidelines could demonstrate significant benefits to outcomes and cost, and the healthcare system over time. Future longitudinal research could help us understand how adherence to these guidelines could improve care and lifespan in people with SCD.

Not only was the primary care provider associated with higher primary care guideline adherence, but having an embedded PCP also had an association with more SCD-specific guideline adherence. There were more screenings for eye exams, protein in urine checks, and, most notably, more individuals with an elevated protein in their urine who are also on critical medications, such as ACE inhibitors or ARBs. Our manuscript is one of the first to demonstrate increased adherence to the recent ASH guidelines, such as screening for proteinuria and being on medications with positive proteinuria when a PCP is embedded in SCD teams. The potential long-term impact of better adherence could be significant in slowing the progression of kidney disease; however, this hypothesis would need to be tested in prospective longitudinal cohorts. This model was primarily for adults; there are additional pediatric guidelines, such as annual transcranial Doppler screening. Future research of an embedded PCP in pediatric SCD clinics could demonstrate better adherence across multiple other SCD-specific guidelines.

Patients seen by the embedded PCP had evolving healthcare utilization. Within the embedded PCP cohort, annual outpatient visits increased after integration, which would be consistent with improved continuity and engagement in longitudinal care. The modest increases in ED visits may indicate more vigilant symptom recognition or more comprehensive inpatient evaluation. While statistically insignificant, differences in inpatient admissions narrowed after the embedded PCP was seen, suggesting that coordinated primary and specialty care may help stabilize hospitalization use, including those higher‑risk patients.Our findings suggest that an embedded PCP may initially reveal unmet medical needs (e.g., uncontrolled diabetes, poorly controlled asthma, moderate to severe depression and anxiety, suicidal ideation, and post-traumatic stress disorder), leading to increasing utilization, while also strengthening self-efficacy and preventive care delivery and setting the foundation for potential long‑term disease management. These hypotheses would need to be tested in future longer longitudinal cohort studies.

Our embedded PCP model may be a cost-effective and scalable approach to SCD-care. There is limited cost overhead as it uses an existing clinical infrastructure and workforce to integrate primary care services into visits that patients are already attending. There may be cost savings due to the potential to reduce duplicate appointments with multiple clinical infrastructure and staff, missed preventive care appointments, and a decrease of costly acute care hospitalizations. Scaling this approach may be feasible through part-time allocation of primary care provider efforts, shared staffing across multiple specialty clinics, and use of EHR-based preventive care dashboards and standardized workflows. However, cost-effectiveness analyses and multi-site implementation studies are needed to quantify return on investment and identify the staffing and infrastructure requirements for broader adoption.

This model of care could be applied to other chronic diseases, as primary care is a challenge to get, especially primary care providers who are knowledgeable about the disease, such as other rare diseases, including hemophilia and other rare hematologic disorders, survivors of childhood cancer, cystic fibrosis, type one diabetes, and epilepsy, among others. In addition, the embedded PCP could also be useful for long-term follow-up clinics after bone marrow transplant or gene therapy for SCD.

This study was limited due to being performed at a single center and had a selection bias of only including those who presented in the SCD clinic, which could limit generalizability. Another limitation is that the sample was not randomized leading to potential selection bias. Some examples of potential limitations of selection bias include reasons as to why some people would not accept the option to receive PCP care on their team, such as not having a PCP, ease of having multiple appointments at one time, knowledgeable PCPs who understand SCD, trust in the PCP as they work with the hematologist that they already have trust in. There are ethical challenges to randomizing patients to obtain improved access to primary care. Future studies with stepped wedge designs could help in evaluation of this model. We evaluated inpatient hospitalizations but were unable to include observation hospitalizations. When patients get an IV pain medication, they are considered inpatient hospitalizations, which is the most common treatment for pain crises requiring hospitalizations; therefore, we likely captured most of the hospitalizations. While we can evaluate many hospitalizations from our Epic EHR, there is a possibility that some patients had outside visits that were not captured. Also, EHR data is limited in its ability to measure medication adherence. These and other confounders, such as changes in care over the 5-year period (e.g., availability of medications, insurance coverages, the pandemic), could be further evaluated in future multi-site implementation studies. Another limitation is that guideline adherence was derived from Epic health-maintenance topics, based on the most recent completion value. Patients seen by the embedded PCP possibly had more opportunities for internal documentation and longer follow-up after integration. While some outside screenings, vaccinations, and eye care were captured similarly between groups (e.g., vaccinations are based on a state registry, so if they did receive them at other primary care facilities within the state they would be captured), some preventive guideline outcomes, such as cervical cancer screening may reflect both care delivery and documentation intensity. Baseline differences between groups may also have influenced the observed outcomes. In particular, the longer follow-up duration in this group may have increased the likelihood of being classified as “up to date” with preventive measures. These factors could partially account for the observed improvement in guideline adherence, independent of the intervention itself.

Our results are consistent with prior literature describing that integrated primary care can be associated with better guideline-directed care and improve outpatient healthcare utilization [42,43]. The literature also suggests a benefit to hospitalization risk when both PCP and hematology are involved, but unlike this study, they did not describe an embedded PCP within the SCD care teams’ model or report detailed completion of general and SCD-specific preventive guidelines. While the focus of this manuscript was on preventive care and how an integrated PCP alongside a hematologist could affect preventive care and healthcare utilization, evaluating models where the PCP manages SCD and inclusion of SCD-specific care and outcomes would be an interesting area of further research

By (1) improving access to PCPs, (2) implementing a team structure that allows a patient-centered medical home to be effectively addressed, and (3) using evidence-based guidelines, we can potentially shift the health paradigm in SCD from a model that is ineffective to a model that emphasizes comprehensive treatment, meets the access and care needs of these vulnerable patients, supports more appropriate healthcare utilization, and improves patient outcomes. Further work at more institutions with embedded PCPs, extending to the pediatric population, and evaluating patient outcomes and cost-effectiveness analyses are potential areas for future research.

## Supporting information

S1 File All Health Maintenance Topics that fall into the Health Maintenance Categories with the “prior time” and “post time” ranges that are referenced in the table above. Prior time means the time before the exact due date that the topic will show as “due soon”. Post time is the time after the exact due date that the topic will show as “overdue”. For example, if a patient is aging into the Colorectal Cancer Screening topic at age 45 and their DOB is 1/1/1981, their exact due date for Colorectal Cancer Screening would be 1/1/2026. The prior and post time for this topic is 1 month, so they would show as “due soon” on 12/1/2025, “due on” from 1/1/2026–1/31/2026, and “overdue” on 2/1/2026. Each Health Maintenance Category depends on the clinical context to determine the appropriate Topic Name. For example, there are multiple frequencies Cervical Cancer Screening as depending on the patient’s age and previous pap smear and HPV testing results, their next Cervical Cancer Screening could be 12 month, 36 months, 60 months, etc. from the previous screening.
(DOCX)

S2 File Definition of Health Maintenance Categories’ Status. (DOCX)

S3 File Patient up to date Compliance at last visit, n = 388. (DOCX)

S4 File Patient up to date Compliance at last visit (at least 2 visits and 365 days FU). (DOCX)

S5 File De-identified data set. (XLSX)
