# Supplementary material for: Embedding a primary care provider in sickle cell teams improves sickle cell care
Source: PLoS One. 2026 Jun 25;21(6):e0352670. doi: 10.1371/journal.pone.0352670 (PMC13298928; doi:10.1371/journal.pone.0352670)
Supplement: S1 File — Prior time means the time before the exact due date that the topic will show as “due soon”. Post time is the time after the exact due date that the topic will show as “overdue”. For example, if a patient is aging into the Colorectal Cancer Screening topic at age 45 and their DOB is 1/1/1981, their exact due date for Colorectal Cancer Screening would be 1/1/2026. The prior and post time for this topic is 1 month, so they would show as “due soon” on 12/1/2025, “due on” from 1/1/2026–1/31/2026, and “overdue” on 2/1/2026. Each Health Maintenance Category depends on the clinical context to determine the appropriate Topic Name. For example, there are multiple frequencies Cervical Cancer Screening as depending on the patient’s age and previous pap smear and HPV testing results, their next Cervical Cancer Screening could be 12 month, 36 months, 60 months, etc. from the previous screening. (DOCX) [file pone.0352670.s001.docx]

| **S1**: All Health Maintenance Topics that fall into the Health Maintenance Categories with the “prior time” and “post time” ranges that are referenced in the table above. Prior time means the time before the exact due date that the topic will show as “due soon”. Post time is the time after the exact due date that the topic will show as “overdue”. For example, if a patient is aging into the Colorectal Cancer Screening topic at age 45 and their DOB is 1/1/1981, their exact due date for Colorectal Cancer Screening would be 1/1/2026. The prior and post time for this topic is 1 month, so they would show as “due soon” on 12/1/2025, “due on” from 1/1/2026-1/31/2026, and “overdue” on 2/1/2026. Each Health Maintenance Category depends on the clinical context to determine the appropriate Topic Name. For example, there are multiple frequencies Cervical Cancer Screening as depending on the patient’s age and previous pap smear and HPV testing results, their next Cervical Cancer Screening could be 12 month, 36 months, 60 months, etc. from the previous screening. | | | | | | |
| --- | --- | --- | --- | --- | --- | --- |
| **Topic ID** | **Topic Name** | **Frequency (months)** | **Health Maintenance Category** | **Prior Time (D30 = 30 days, W2 = 2 weeks, 1 = 1 month)** | **Post Time** | **Grace Time** |
| 13 | CERVICAL CANCER SCREENING DISCUSSION | 12 | Cervical Cancer Screening | 1 | 1 |  |
| 1051 | HPV TEST | 6 | Cervical Cancer Screening |  |  |  |
| 1053 | HPV TEST | 12 | Cervical Cancer Screening |  |  |  |
| 1054 | HPV TEST | 24 | Cervical Cancer Screening |  |  |  |
| 1055 | HPV TEST | 36 | Cervical Cancer Screening |  |  |  |
| 72 | HPV TEST | 60 | Cervical Cancer Screening |  |  |  |
| 5050 | PAP SMEAR | 6 | Cervical Cancer Screening | 1 | 1 |  |
| 5051 | PAP SMEAR | 24 | Cervical Cancer Screening | 1 | 1 |  |
| 5052 | PAP SMEAR | 36 | Cervical Cancer Screening | 1 | 1 |  |
| 6001 | PAP SMEAR | 6 | Cervical Cancer Screening | 1 | 1 |  |
| 6002 | PAP SMEAR | 12 | Cervical Cancer Screening | 1 | 1 |  |
| 6003 | PAP SMEAR | 24 | Cervical Cancer Screening | 1 | 1 |  |
| 6004 | PAP SMEAR | 36 | Cervical Cancer Screening | 1 | 1 |  |
| 6005 | PAP SMEAR | 48 | Cervical Cancer Screening | 1 | 1 |  |
| 6006 | PAP SMEAR | 60 | Cervical Cancer Screening | 1 | 1 |  |
| 5080 | PAP SMEAR | 48 | Cervical Cancer Screening | 1 | 1 |  |
| 5081 | PAP SMEAR | 60 | Cervical Cancer Screening | 1 | 1 |  |
| 5083 | PAP SMEAR | 12 | Cervical Cancer Screening | 1 | 1 |  |
| 6000 | PAP SMEAR DISCUSSION | 12 | Cervical Cancer Screening | 1 | 1 |  |
| 24 | CHLAMYDIA SCREEN | 12 | Chlamydia Screening | 1 | 1 |  |
| 82 | COLONOSCOPY | 84 | Colorectal Cancer Screening | 1 | 1 |  |
| 55 | COLONOSCOPY | 24 | Colorectal Cancer Screening | 1 | 1 |  |
| 5008 | COLONOSCOPY | 48 | Colorectal Cancer Screening | 1 | 1 |  |
| 16 | COLONOSCOPY | 120 | Colorectal Cancer Screening | 1 | 1 |  |
| 45 | COLONOSCOPY | 60 | Colorectal Cancer Screening | 1 | 1 |  |
| 46 | COLONOSCOPY | 36 | Colorectal Cancer Screening | 1 | 1 |  |
| 47 | COLONOSCOPY | 12 | Colorectal Cancer Screening | 1 | 1 |  |
| 44 | COLORECTAL CANCER SCREENING DISCUSSION | 12 | Colorectal Cancer Screening | 1 | 1 |  |
| 41 | DOUBLE CONTRAST BARIUM ENEMA | 60 | Colorectal Cancer Screening |  |  |  |
| 21 | FECAL IMMUNOCHEMICAL TEST | 12 | Colorectal Cancer Screening |  |  |  |
| 43 | FECAL OCCULT BLOOD TEST | 12 | Colorectal Cancer Screening | 1 | 1 |  |
| 59 | FLEXIBLE SIGMOIDOSCOPY | 60 | Colorectal Cancer Screening | 1 | 1 |  |
| 5000 | STOOL DNA | 36 | Colorectal Cancer Screening | D30 | D30 |  |
| 42 | TOMOGRAPHIC COLONOGRAPHY | 60 | Colorectal Cancer Screening | 1 | 1 |  |
| 85 | DEPRESSION SCREENING | 12 |  | 1 | 1 |  |
| 5084 | DTAP/TDAP/TD VACCINE | -90 | Diphtheria, Tetanus, and Pertussis Immunization |  | 18 | D4 |
| 5057 | TDAP (ADULT) | -1 | Diphtheria, Tetanus, and Pertussis Immunization | 1 | 1 |  |
| 17 | TETANUS | 120 | Diphtheria, Tetanus, and Pertussis Immunization | 1 | 1 |  |
| 5063 | EYE EXAM | 12 | Eye Exam | 1 | 1 |  |
| 5082 | GLAUCOMA SCREENING | 12 | Eye Exam |  |  |  |
| 69 | EYE EXAM | 24 | Eye Exam | 1 | 1 |  |
| 5076 | HIV SCREENING DISCUSSION | -1 | HIV Screening | 1 | 1 |  |
| 5085 | HPV VACCINE | -90 | HPV Immunization |  | 12 | D4 |
| 30 | HPV VACCINE ADOL | -90 | HPV Immunization |  | 12 | D4 |
| 28 | HEP B VACCINE | -90 | Hepatitis B Immunization |  | D30 | D4 |
| 5074 | HEPATITIS C VIRUS SCREENING | -1 | Hepatitis C Screening | 1 | 1 |  |
| 20 | INFLUENZA VACCINE | -90 | Influenza Immunization | W2 | W1 | D7 |
| 5019 | LIPID SCREENING | 6 | Lipid Panel | 1 | 1 |  |
| 5020 | LIPID SCREENING | 3 | Lipid Panel | 1 | 1 |  |
| 5013 | LIPID SCREENING | 60 | Lipid Panel | 1 | 1 |  |
| 5015 | LIPID SCREENING | 24 | Lipid Panel | 1 | 1 |  |
| 5017 | LIPID SCREENING | 12 | Lipid Panel | 1 | 1 |  |
| 60 | LIPIDS | 12 | Lipid Panel | 1 | 1 |  |
| 51 | MAMMOGRAM | 12 | Mammography | 1 | 1 |  |
| 52 | MAMMOGRAM | 24 | Mammography | 1 | 1 |  |
| 53 | MAMMOGRAM | 60 | Mammography | 1 | 1 |  |
| 5006 | MAMMOGRAM | 6 | Mammography | 1 | 1 |  |
| 6015 | MAMMOGRAM | 12 | Mammography | 1 | 1 |  |
| 6016 | MAMMOGRAM | 12 | Mammography | 1 | 1 |  |
| 6017 | MAMMOGRAM | 12 | Mammography | 1 | 1 |  |
| 6018 | MAMMOGRAM | 12 | Mammography | 1 | 1 |  |
| 25 | MAMMOGRAM LEFT | 12 | Mammography | 1 | 1 |  |
| 23 | MAMMOGRAM RIGHT | 12 | Mammography | 1 | 1 |  |
| 6014 | MAMMOGRAM SCREENING DISCUSSION | 12 | Mammography | 1 | 1 |  |
| 14 | MAMMOGRAM SCREENING DISCUSSION | 12 | Mammography | 1 | 1 |  |
| 31 | MCV4 VACCINE | -90 | Meningococcal ACWY Immunization |  | 1 | D4 |
| 38 | MENINGOCOCCAL B VACCINE | -90 | Meningococcal B Immunization | 1 |  | D4 |
| 50 | PNEUMOCOCCAL VACCINE SERIES | -90 | Pneumococcal Immunization | 1 | 1 | D4 |
| 36 | PNEUMOCOCCAL VACCINE SERIES | -90 | Pneumococcal Immunization |  | D30 | D4 |
| 5003 | PROSTATE CANCER SCREENING DISCUSSION | 12 | Prostate Specific Antigen (PSA) Screening | 1 | 1 |  |
| 5018 | PSA | 12 | Prostate Specific Antigen (PSA) Screening | 1 | 1 |  |
| 5075 | PSA | 24 | Prostate Specific Antigen (PSA) Screening |  |  |  |
| 5009 | PSA | 6 | Prostate Specific Antigen (PSA) Screening |  |  |  |
