# Supplementary material for: Embedding a primary care provider in sickle cell teams improves sickle cell care
Source: PLoS One. 2026 Jun 25;21(6):e0352670. doi: 10.1371/journal.pone.0352670 (PMC13298928; doi:10.1371/journal.pone.0352670)
Supplement: S2 File — (DOCX) [file pone.0352670.s002.docx]

| **S2:** Definition of Health Maintenance Categories’ Status | |
| --- | --- |
| **Status** | **Definition** |
| Not Due | The task has been done for this frequency |
| Due Soon | Task is due within the prior time set in the topic's record |
| Due On | Task's due date has passed, but the post-time has not |
| Overdue | Task's post-time has passed |
| Postponed | A user has postponed the topic |
| Completed | Only used with a frequency of "Once" or for sequential topics |
| Addressed | Used in immunization sequences if the series has been completed by an override |
| Aged Out | The topic was not completed, but the patient is no longer eligible |
| Discontinued | The patient has been removed from the topic by a user |
| Hidden | The patient qualifies for the topic, but the start date has not been reached yet |
