# Supplementary material for: Embedding a primary care provider in sickle cell teams improves sickle cell care
Source: PLoS One. 2026 Jun 25;21(6):e0352670. doi: 10.1371/journal.pone.0352670 (PMC13298928; doi:10.1371/journal.pone.0352670)
Supplement: S3 File — (DOCX) [file pone.0352670.s003.docx]

| **S3. Patient up to date Compliance at last visit, n=388** | | | | | |
| --- | --- | --- | --- | --- | --- |
| **Outcomes (N observed/N available, %)** | **Total  (n=388)** | **Non-PCP (n=214)** | **PCP (n=174)** | **OR (95% CI)** | **P-value** |
| Cervical Cancer Screening | 92/213 (43.19) | 38/127 (29.92) | 54/86 (62.79) | 4.49 (2.46, 8.23) | <.0001 |
| Chlamydia Screening | 27/49 (55.10) | 11/28 (39.29) | 16/21 (76.19) | 5.43 (1.48, 19.93) | 0.0107 |
| Colorectal Cancer Screening | 29/89 (32.58) | 17/56 (30.36) | 12/33 (36.36) | 2.37 (0.75, 7.47) | 0.1417 |
| Depression Screening | 329/346 (95.09) | 160/175 (91.43) | 169/171 (98.83) | 7.97 (1.78, 35.69) | 0.0002 |
| Diphtheria, Tetanus, and Pertussis Immunization | 287/387 (74.16) | 142/214 (66.36) | 145/173 (83.82) | 2.88 (1.72, 4.84) | <.0001 |
| Eye Exam | 168/349 (48.14) | 65/178 (36.52) | 103/171 (60.23) | 2.59 (1.66, 4.04) | <.0001 |
| HIV Screening | 298/377 (79.05) | 138/208 (66.35) | 160/169 (94.67) | 10.43 (4.93, 22.07) | <.0001 |
| HPV Immunization | 82/148 (55.41) | 34/69 (49.28) | 48/79 (60.76) | 2.38 (1.13, 5.01) | 0.0230 |
| Hepatitis B Immunization | 93/180 (51.67) | 35/80 (43.75) | 58/100 (58.00) | 1.55 (0.72, 3.31) | 0.2622 |
| Hepatitis C Screening | 308/381 (80.84) | 142/207 (68.60) | 166/174 (95.40) | 11.52 (5.21, 25.45) | <.0001 |
| Influenza Immunization | 298/380 (78.42) | 160/213 (75.12) | 138/167 (82.63) | 1.72 (1.02, 2.91) | 0.0418 |
| Lipid Panel | 94/147 (63.95) | 43/94 (45.74) | 51/53 (96.23) | 37.03 (8.22, 166.74) | <.0001 |
| Mammography | 34/70 (48.57) | 22/46 (47.83) | 12/24 (50.00) | 1.06 (0.37, 3.07) | 0.9143 |
| Meningococcal ACWY Immunization | 121/347 (34.87) | 48/176 (27.27) | 73/171 (42.69) | 2.02 (1.27, 3.20) | 0.0031 |
| Meningococcal B Immunization | 24/342 (7.02) | 11/173 (6.36) | 13/169 (7.69) | 1.09 (0.47, 2.56) | 0.8412 |
| Pneumococcal Immunization | 207/322 (64.29) | 83/162 (51.23) | 124/160 (77.50) | 3.58 (2.16, 5.92) | <.0001 |
| Prostate Specific Antigen (PSA) Screening | 7/18 (38.89) | 5/15 (33.33) | 2/3 (66.67) | 2.71 (0.11, 67.47) | 0.5432 |
| Urine Protein Screening | 273/348 (78.45) | 123/178 (69.10) | 150/170 (88.24) | 3.36 (1.89, 6.00) | <.0001 |
| On ACE/ARB | 48/90 (53.33) | 14/44 (31.82) | 34/46 (73.91) | 9.37 (3.11, 28.23) | <.0001 |
