# Supplementary material for: Embedding a primary care provider in sickle cell teams improves sickle cell care
Source: PLoS One. 2026 Jun 25;21(6):e0352670. doi: 10.1371/journal.pone.0352670 (PMC13298928; doi:10.1371/journal.pone.0352670)
Supplement: S4 File — (DOCX) [file pone.0352670.s004.docx]

| **S4: Patient up to date Compliance at last visit (at least 2 visits and 365 days FU)** | | | | | |
| --- | --- | --- | --- | --- | --- |
| **Outcomes (N observed/N available, %)** | **Total  (n=260)** | **Non-PCP (n=133)** | **PCP (n=127)** | **OR (95% CI)** | **P-value** |
| Cervical Cancer Screening | 72/142 (50.70) | 31/80 (38.75) | 41/62 (66.13) | 3.31 (1.62, 6.75) | 0.0010 |
| Chlamydia Screening | 15/25 (60.00) | 7/15 (46.67) | 8/10 (80.00) | 5.69 (0.82, 39.22) | 0.0777 |
| Colorectal Cancer Screening | 22/64 (34.38) | 13/39 (33.33) | 9/25 (36.00) | 2.32 (0.59, 9.04) | 0.2262 |
| Depression Screening | 244/253 (96.44) | 119/126 (94.44) | 125/127 (98.43) | 3.89 (0.78, 19.51) | 0.0990 |
| Diphtheria, Tetanus, and Pertussis Immunization | 211/259 (81.47) | 101/133 (75.94) | 110/126 (87.30) | 2.21 (1.11, 4.40) | 0.0248 |
| Eye Exam | 119/253 (47.04) | 49/126 (38.89) | 70/127 (55.12) | 1.78 (1.06, 2.98) | 0.0288 |
| HIV Screening | 223/254 (87.80) | 99/129 (76.74) | 124/125 (99.20) | 41.80 (5.52, 316.48) | 0.0003 |
| HPV Immunization | 57/98 (58.16) | 23/40 (57.50) | 34/58 (58.62) | 1.71 (0.64, 4.58) | 0.2832 |
| Hepatitis B Immunization | 70/132 (53.03) | 25/56 (44.64) | 45/76 (59.21) | 1.38 (0.58, 3.31) | 0.4708 |
| Hepatitis C Screening | 226/259 (87.26) | 102/132 (77.27) | 124/127 (97.64) | 12.79 (3.74, 43.76) | <.0001 |
| Influenza Immunization | 205/252 (81.35) | 105/132 (79.55) | 100/120 (83.33) | 1.50 (0.77, 2.92) | 0.2295 |
| Lipid Panel | 73/105 (69.52) | 36/67 (53.73) | 37/38 (97.37) | 35.70 (4.52, 281.94) | 0.0007 |
| Mammography | 27/51 (52.94) | 17/32 (53.13) | 10/19 (52.63) | 1.14 (0.35, 3.71) | 0.8330 |
| Meningococcal ACWY Immunization | 96/253 (37.94) | 39/126 (30.95) | 57/127 (44.88) | 1.72 (1.01, 2.93) | 0.0445 |
| Meningococcal B Immunization | 15/250 (6.00) | 7/125 (5.60) | 8/125 (6.40) | 0.91 (0.31, 2.67) | 0.8567 |
| Pneumococcal Immunization | 159/225 (70.67) | 65/109 (59.63) | 94/116 (81.03) | 2.89 (1.54, 5.42) | 0.0010 |
| Prostate Specific Antigen (PSA) Screening | 5/13 (38.46) | 4/11 (36.36) | 1/2 (50.00) | 2.66 (0.08, 86.27) | 0.5808 |
| Urine Protein Screening | 205/252 (81.35) | 93/126 (73.81) | 112/126 (88.89) | 2.92 (1.44, 5.93) | 0.0031 |
| On ACE/ARB | 41/75 (54.67) | 12/39 (30.77) | 29/36 (80.56) | 15.86 (3.85, 65.31) | 0.0001 |
